# Supplementary material for: Prevalence, socio-demographic and environmental determinants of asthma in 4621 Ghanaian adults: Evidence from Wave 2 of the World Health Organization’s study on global AGEing and adult health
Source: PLoS One. 2020 Dec 9;15(12):e0243642. doi: 10.1371/journal.pone.0243642 (PMC7725342; doi:10.1371/journal.pone.0243642)
Supplement: Attachment — Submitted filename: Response_to_Reviewers_Editors_JA.docx [file pone.0243642.s001.docx]

**Responses from the authors:**

First, we extend our profound gratitude and thanks to the Editors and the Reviewers for their efforts and valuable comments on our paper.

We have given your comments the needed attention and action and are happy to resubmit the revised manuscript for your kind consideration and subsequent publication in your cherished journal, and together we can all help in addressing this serious public health challenge facing millions of people in most developing countries like Ghana.

*Below are our responses to the comments in italics and you will also find the needed changes in the main report tracked in colour using the tracking facility in word. In addition, we have made few other changes to the paper with the sole aim of enhancing the understanding of our readers and these were also tracked in colour.*

**Journal Requirements:**

When submitting your revision, we need you to address these additional requirements.

1. Please ensure that your manuscript meets PLOS ONE's style requirements, including those for file naming. The PLOS ONE style templates can be found at

https://journals.plos.org/plosone/s/file?id=wjVg/PLOSOne_formatting_sample_main_body.pdf and

https://journals.plos.org/plosone/s/file?id=ba62/PLOSOne_formatting_sample_title_authors_affiliations.pdf

***Response:*** *we have revised the manuscript to address this (e.g. see the tittle page, abstract and the introduction, materials and methods).*

2. Thank you for stating the following in the Acknowledgments Section of your manuscript:

"Financial support was provided by the US National Institute on Aging through
Interagency Agreements (OGHA 04034785; YA1323-08-CN-0020; Y1-AG-1005-01) with the
World Health Organization and a Research Project Grant (R01 AG034479- 64401A1). WHO
contributed financial and human resources to SAGE. The Ministry of Health, Ghana, is
supportive of SAGE. The University of Ghana’s Department of Community Health contributed
training facilities, data entry support, and storage of materials. The Ghana Statistical Office
provided the sampling information for the sampling frame and updates."

We note that you have provided funding information that is not currently declared in your Funding Statement. However, funding information should not appear in the Acknowledgments section or other areas of your manuscript. We will only publish funding information present in the Funding Statement section of the online submission form.

Please remove any funding-related text from the manuscript and let us know how you would like to update your Funding Statement. Currently, your Funding Statement reads as follows:

"The authors received no specific funding for this work"

***Response:*** *Thank you. The present study (the work in this manuscript) did not receive any funding. We were only acknowledging those who funded the original survey that generated the data which is serving as a secondary data for the present study. However, some of my co-authors were part of investigators during the main survey. We revised the manuscript by removing the funding statement under the acknowledgement section and also removed the ‘funding section’ from the manuscript (see lines 7-12, page 20).*

*The acknowledgement now reads “We are grateful to the World Health Organization and her partners who made the SAGE study possible and provided us with the data at no cost. We are also thankful to all respondents and interviewers who participated in the SAGE survey in Ghana. The Ministry of Health, Ghana, is supportive of SAGE. The University of Ghana’s Department of Community Health contributed training facilities, data entry support, and storage of materials. The Ghana Statistical Service provided the sampling information for the sampling frame and updates.”*

*For the online funding statement, the following can be used or you can modify it to suit the journal requirement: “Funding was obtained for the main WHO SAGE Wave 2 Survey in Ghana with financial support provided by the US National Institute on Aging through Interagency Agreements (OGHA 04034785; YA1323-08-CN-0020; Y1-AG-1005-01) with the World Health Organization and a Research Project Grant (R01 AG034479- 64401A1). The funder provided support in the form of salaries for authors [RB, AEY], but did not have any additional role in the study design, data collection and analysis, decision to publish, or preparation of the manuscript. The specific roles of these authors are articulated in the ‘author contributions’ section. However, the work in this study did not receive any funding support. Also, Deloitte Consulting, West Africa Deloitte & Touche did not provide any financial support for the study and played no role in the study”. See response to point 3 below:*

3. Thank you for stating the following in the Competing Interests section:

"The authors have declared that no competing interests exist."

We note that one or more of the authors are employed by a commercial company: 3Deloitte Consulting, West Africa Deloitte & Touche.

***Response:*** *Thank you. Deloitte Consulting, West Africa Deloitte & Touche did not provide any financial support for the study and played no role in the study. Our co-author, Mr Kallson participated in this study in his capacity as an individual. Thus, he was not representing his company. We addressed this in our response to point 2 above.*

3.1. Please provide an amended Funding Statement declaring this commercial affiliation, as well as a statement regarding the Role of Funders in your study. If the funding organization did not play a role in the study design, data collection and analysis, decision to publish, or preparation of the manuscript and only provided financial support in the form of authors' salaries and/or research materials, please review your statements relating to the author contributions, and ensure you have specifically and accurately indicated the role(s) that these authors had in your study. You can update author roles in the Author Contributions section of the online submission form.

Please also include the following statement within your amended Funding Statement.

“The funder provided support in the form of salaries for authors [insert relevant initials], but did not have any additional role in the study design, data collection and analysis, decision to publish, or preparation of the manuscript. The specific roles of these authors are articulated in the ‘author contributions’ section.”

If your commercial affiliation did play a role in your study, please state and explain this role within your updated Funding Statement.

***Response:*** *Thank you. The commercial affiliation did not play any role in the study. We have updated the funding (We addressed this in our response to point 2 above) and the author contribution statements (lines 13-16, page 19) to reflect the changes required.*

We provided the following: *“All authors except Mr Kallson declare that they have no conflict of interest. Though Mr Kallson was working with Deloitte Consulting, West Africa Deloitte & Touche at the time of the study, Deloitte Consulting did not provide any financial support for the study and did not play any role in the study design, data collection and analysis, preparation of the manuscript, or decision to publish. Mr Kallson participated in this study in his capacity as an individual and was not acting on behalf of Deloitte Consulting, and this does not alter our adherence to PLOS ONE policies on sharing data and materials.”*

3.2. Please also provide an updated Competing Interests Statement declaring this commercial affiliation along with any other relevant declarations relating to employment, consultancy, patents, products in development, or marketed products, etc.

Within your Competing Interests Statement, please confirm that this commercial affiliation does not alter your adherence to all PLOS ONE policies on sharing data and materials by including the following statement: "This does not alter our adherence to  PLOS ONE policies on sharing data and materials.” (as detailed online in our guide for authors [http://journals.plos.org/plosone/s/competing-interests](about:blank)) . If this adherence statement is not accurate and  there are restrictions on sharing of data and/or materials, please state these. Please note that we cannot proceed with consideration of your article until this information has been declared.

Please include both an updated Funding Statement and Competing Interests Statement in your cover letter. We will change the online submission form on your behalf.

Please know it is PLOS ONE policy for corresponding authors to declare, on behalf of all authors, all potential competing interests for the purposes of transparency. PLOS defines a competing interest as anything that interferes with, or could reasonably be perceived as interfering with, the full and objective presentation, peer review, editorial decision-making, or publication of research or non-research articles submitted to one of the journals. Competing interests can be financial or non-financial, professional, or personal. Competing interests can arise in relationship to an organization or another person. Please follow this link to our website for more details on competing interests: [http://journals.plos.org/plosone/s/competing-interests](about:blank)

***Response:*** *Thank you. We have provided the updated funding and competing interests statement in the cover letter as requested.*

4. We note that you have indicated that data from this study are available upon request. PLOS only allows data to be available upon request if there are legal or ethical restrictions on sharing data publicly. For information on unacceptable data access restrictions, please see http://journals.plos.org/plosone/s/data-availability#loc-unacceptable-data-access-restrictions.

In your revised cover letter, please address the following prompts:

a) If there are ethical or legal restrictions on sharing a de-identified data set, please explain them in detail (e.g., data contain potentially identifying or sensitive patient information) and who has imposed them (e.g., an ethics committee). Please also provide contact information for a data access committee, ethics committee, or other institutional body to which data requests may be sent.

***Response:*** *There is no legal restrictions on the data known to the authors. The data is provided to researchers freely but the WHO SAGE Team only releases the data to researchers upon request directly made to WHO, and individual researchers granted permission to use the data are not allowed to make the data (in any form) available to third parties. Any third party interested in the data must apply directly to the WHO SAGE Team. We have included this in the cover letter as requested, and also in the manuscript.*

*Under data availability, we inserted “Data is freely available upon making official request to WHO SAGE Team through the WHO website at* [*http://www.who.int/healthinfo/sage/cohorts/en/*](http://www.who.int/healthinfo/sage/cohorts/en/)*. The data is provided to researchers freely but the WHO SAGE Team only releases the data to researchers upon request made directly to WHO, and individual researchers granted permission to use the data are not allowed to make the data (in any form) available to third parties. Any third party interested in the data must apply directly to the WHO SAGE Team”. See lines 1-2, page 19 and lines 1-4, page 20.*

b) If there are no restrictions, please upload the minimal anonymized data set necessary to replicate your study findings as either Supporting Information files or to a stable, public repository and provide us with the relevant URLs, DOIs, or accession numbers. Please see http://www.bmj.com/content/340/bmj.c181.long for guidelines on how to de-identify and prepare clinical data for publication. For a list of acceptable repositories, please see http://journals.plos.org/plosone/s/data-availability#loc-recommended-repositories.

We will update your Data Availability statement on your behalf to reflect the information you provide.

***Response:*** *Please, refer to our response to point 4(a) and also to the cover letter.*

**Reviewers' comments:**

Reviewer #1: I felt this was a well done study. The manuscript provides helpful information on Ghanaian health that contains actionable areas for future public health interventions. The addition of a) a map of Ghana, and b) more geographical context for from where within Ghana respondents live would strengthen the paper.

***Response:*** *Thank you for the useful comment. Unfortunately, the survey did not collect data on spatial coordinates (latitude and longitude) of the participants in the 2014/2015 study. Our current study is based on this secondary data and it is therefore not possible to show the locations of these participants on the map of Ghana in the present study.*

Reviewer #2: This is an exploratory study examining the prevalence, socio-demographic and environmental determinants of asthma in Ghana. My main concerns include 1) the cross-sectional design, 2) the lack of considerations of neighborhood-level risk factors, and 3) the variable selection procedure used. Below please find my detailed comments.

1. Page 3, background, reference 1: please update the reference and cite the most recent statistics from the global burden of disease study.

***Response:*** *Thank you. Revised as suggested.* *In the 2020 GBD study report, there is no combine prevalence and number of deaths for asthma and chronic obstructive pulmonary disease. As a result, we revised the text and provide the updated statistics from the 2020 report while maintaining the 2017 statistics also and both referenced. See lines 2-8 at page 3.*

2. Page 5, study population: please briefly describe the sampling strategy used in SAGE.

***Response:*** *Thanks for the useful feedback. We revised the manuscript to reflect this.* *However, we effected this under data source and sampling because we believe is more appropriate under the data source compared to the study population. See line 14-20 at page 5.*

3. Page 5, study population and outcome variable: since SAGE is a longitudinal study, it might be better to use data from both wave 1 and wave 2 to identify incident asthma cases. The current analsyes depending on the asthma question only from the wave 2 made this a cross-sectional study, which substantially limited the significance of this study.

***Response:*** *The focus of this paper is to quantify unobserved household-level residual variations in asthma episodes while simultaneously identifying critical risk factors associated with the disease. We agreed that using data from wave 1 and 2 might provide additional information BUT there was a challenge where over 50% of those who participated in wave 1 conducted in 2007-2008 dropped out of the study in wave 2 conducted in 2014-2015 and new samples were drawn to replace some of them. Also, the secondary data we used did not uniquely identify participants in wave 1 only, wave 2 only and those in both wave 1 and 2. This introduced some analytical challenges because in analysing both wave 1 and wave 2, the modelling become much complex because we need to take into account that majority of those in wave 1 did not participate in wave 2 and that new samples were introduced in wave 2. Thus, there is a mixture of repeated cross-sectional and longitudinal designs which must be considered when modelling based on wave 1 and 2 but this will require availability of further data (e.g. variables that uniquely identify participants in wave 1 only, wave 2 only and those in both wave 1 and 2) and also this kind of analysis will require much more efforts than that of the present study. We will continue to engage the owners of the data and if successful, we will implement a modelling strategy that will account for the mixture of repeated cross-sectional and longitudinal designs nature of the data in our future studies.*

4. Page 5, explanatory variables: as this is an exploratory study, it is unclear why the authors only included these variables. Why neighborhood-level factors were not included? Many established risk factors mentioned in the background section were not included.

***Response:*** *We used secondary data in our analysis and except place of residence (urban/rural), no other neighbourhood/environmental covariates were available in the data. If the data were to contain geographical coordinates (latitudes/longitudes) of the respondents (this was not collected in the study), we could use that to extract neighbourhood/environmental/spatial covariates and include same in our model. Also, we explored all potential covariates in the available data through a combination of expert opinion, available literature, and statistical methodology for variable selection.*

5. Page 7, statistical analyses: the authors only included variables with a p-value<0.2 from the univariable regression model in the multivariable regression model. This variable selection approach is problematic. More advanced approach such as the elastic net model or Lasso model with hyperparameters determined by cross-validations should be used to perform the variable selection. e.g., the glmmLasso package in R can be used to implement Lasso for gelearized linear mixed models.

***Response:*** *Thank you for this suggestion.* *First of all,* *we used expert opinion and available literature on deciding potential predictors of asthma episodes to arrive at all the variables presented in Table 1. After that, we applied the statistical methodology for variable selection using p<0.2 in the univariable (bivariate) analysis as a threshold to identify candidate set of variables that will enter the multivariable model which is a standard approach. We will consider the elastic net model or Lasso model suggestion in our future research.*

6. Page 15: one major limitation is the cross-sectional design of this study: the current exposures to the risk factors may not be representative of the historical exposures before the onset of asthma. This needs to be discussed in the limitation section. Again, if asthma data is available from wave 1, I highly recommend the authors to use the data to identify incident asthma cases to avoid the cross-sectional design of the study.

***Response:*** *We already stated in the limitation that the nature of the data and the modelling approach could not allow for drawing effect of causation (see lines 19-20 at page 18), and we did not attempt to make any statement to suggest causation effect between the covariates and asthma episodes. We already provided response to the use of wave 1 and 2 in comment 3 which also addressed this.*
