# Supplementary material for: Prevalence, socio-demographic and environmental determinants of asthma in 4621 Ghanaian adults: Evidence from Wave 2 of the World Health Organization’s study on global AGEing and adult health
Source: PLoS One. 2020 Dec 9;15(12):e0243642. doi: 10.1371/journal.pone.0243642 (PMC7725342; doi:10.1371/journal.pone.0243642)
Supplement: Attachment — Submitted filename: Rebuttal_Letter_Reviewers_JA_AEY_23.11.2020.docx [file pone.0243642.s002.docx]

**Review Comments to the Author**

Reviewer #1: The manuscript has been improved and strengthened. I have rarely seen data on asthma in an African country and this work will add to that literature.

**Response:** Thank you.

Reviewer #2: The authors did not adequately address my concerns, especially for the variable selection procedure. The response letter includes a statement that "we applied the statistical methodology for variable selection using p<0.2 in the univariable (bivariate) analysis as a threshold to identify candidate set of variables that will enter the multivariable model which is a standard approach", which is not true.

**Response:** Thank you for pointing this out. Our decision to use threshold of a p-value <0.2 is based on the discussion in the literature on traditional stopping rule and suggested optimal p-values. The literature on this topic provided a strong recommendation for using a p-value in the range of 0.15–0.20 (Hosmer & Lemeshow, 2013), although using a higher significance level has the disadvantages that some unimportant variables may be included in the model (Hosmer & Lemeshow, 2013). Typically, the stopping rule for the traditional choice for significance level is considered to be between 0.05 and 0.10 though it was also established that the optimum value of the significance level to decide which variable to include in the multiple regression model is suggested to be p<1 (Chowdhury & Turin, 2020; Harrell, 2001), which actually exceeds the traditional choices.

The above discussion in the literature is what informed our choice and that is what the authors meant by the statement "we applied the statistical methodology for variable selection using p<0.2 in the univariable (bivariate) analysis as a threshold to identify candidate set of variables that will enter the multivariable model which is a standard approach". Our choice of p-value<0.2 also lies between the maximum for the traditional rule and that of suggested p-value<1 which is an acceptable approach.

After extensive reading, we observed that there are other techniques such as Lasso, Elastic net, and Ridge regression approaches based on machine learning techniques which could provide optimal variable selection which we did not do in our current study. However, this machine learning approaches of variable selection requires extensive learning of the procedure and the software for implementation, and the authors will surely explore this in their future research work.

We revised the manuscript to highlight this under the limitations in lines 400-403 at page 18. Specifically, we inserted the statement “The use of p<0.2 as the stopping rule to identify candidate set of covariates in the bivariate model to be included in the multivariable model might not provide optimal variable selection for the covariates, although previous studies have provided a strong recommendation for using p-values in the range of 0.15-0.20 [58] as used in our study”.

**References**

Hosmer DW, Lemeshow S, Sturdivant RX. *Applied logistic regression*. New York: John Wiley & Sons, Incorporated, 2013.

Chowdhury MZI, Turin TC. Variable selection strategies and its importance in clinical prediction modelling. Fam Med Community Health. 2020 Feb 16;8(1):e000262. doi: 10.1136/fmch-2019-000262. PMID: 32148735; PMCID: PMC7032893.

Harrell FE. Regression modeling strategies with applications to linear models, logistic regression, and survival analysis. New York: Springer, 2001.
